# Increasing trends in the prevalence of prior cancer in newly diagnosed lung, stomach, colorectal, breast, cervical, and corpus uterine cancer patients: a population-based study

**DOI:** 10.1186/s12885-021-08011-3

**Published:** 2021-03-10

**Authors:** Akira Sato, Keisuke Matsubayashi, Toshitaka Morishima, Kayo Nakata, Koji Kawakami, Isao Miyashiro

**Affiliations:** 1 Department of Cancer Strategy, Cancer Control Center, Osaka International Cancer Institute, 3-1-69 Otemae, Chuo-ku, Osaka, Japan; 2 Department of Pharmacoepidemiology, Graduate School of Medicine and Public Health, Kyoto University, Yoshidakonoe-cho, Sakyo-ku, Kyoto, 606-8501 Japan

**Keywords:** Prior cancer, Cancer survivors, Cancer registry, Prevalence, Second cancer

## Abstract

**Background:**

Cancer survivors are frequently excluded from clinical research, resulting in their omission from the development of many cancer treatment strategies. Quantifying the prevalence of prior cancer in newly diagnosed cancer patients can inform research and clinical practice. This study aimed to describe the prevalence, characteristics, and trends of prior cancer in newly diagnosed cancer patients in Japan.

**Methods:**

Using Osaka Cancer Registry data, we examined the prevalence, characteristics, and temporal trends of prior cancer in patients who received new diagnoses of lung, stomach, colorectal, female breast, cervical, and corpus uterine cancer between 2004 and 2015. Site-specific prior cancers were examined for a maximum of 15 years before the new cancer was diagnosed. Temporal trends were evaluated using the Cochran-Armitage trend test.

**Results:**

Among 275,720 newly diagnosed cancer patients, 21,784 (7.9%) had prior cancer. The prevalence of prior cancer ranged from 3.3% (breast cancer) to 11.1% (lung cancer). In both sexes, the age-adjusted prevalence of prior cancer had increased in recent years (*P* values for trend < 0.001), especially in newly diagnosed lung cancer patients. The proportion of smoking-related prior cancers exceeded 50% in patients with newly diagnosed lung, stomach, colorectal, breast, and cervical cancer.

**Conclusions:**

The prevalence of prior cancer in newly diagnosed cancer patients is relatively high, and has increased in recent years. Our findings suggest that a deeper understanding of the prevalence and characteristics of prior cancer in cancer patients is needed to promote more inclusive clinical research and support the expansion of treatment options.

**Supplementary Information:**

The online version contains supplementary material available at 10.1186/s12885-021-08011-3.

## Background

Cancer survivors are at risk of developing second primary cancers, and their numbers are increasing worldwide [1–5]. A study from the US Surveillance, Epidemiology, and End Results Program reported that almost 20% of individuals with incident cancers between 2009 and 2013 had prior cancer [6]. The number of patients with second malignant cancers is reportedly increasing, which may be influenced by hereditary and familial risk, antecedent cancer therapy, lifestyle-related factors (e.g., tobacco and alcohol consumption), and environmental factors [7].

Cancer survivors require unique medical and psychosocial support with proactive assessments and follow-up care [4]. Several studies have reported that prior cancer did not adversely affect survival in lung and pancreatic cancer patients [8–11]. In contrast, another study noted that poorer survival was associated with some prior cancer types (e.g., colorectum, melanoma, and breast), but not others (e.g., esophagus, stomach, and lung) [12].

There is a growing need for evidence-based strategies to improve the quality and effectiveness of care for cancer survivors. However, the frequent exclusion of cancer survivors from clinical studies may undermine the generalizability of findings on the effectiveness and safety of treatment regimens. Therefore, clarifying the types and trends of prior cancers may help to inform the reevaluation of criteria for participation in clinical research and contribute to the improvement of cancer care [13].

Insight into the prevalence and characteristics of prior cancer can inform research and clinical practice, but few studies have used population-based cancer registry data to examine these issues. In addition, most epidemiological evidence for the association between prior cancer and newly diagnosed cancer is derived from studies conducted in the US and Europe, with little evidence from Asian populations. Extensions to the survival time of cancer patients elevates their risk of developing a second cancer, which could eventually manifest as an increase in the prevalence of cancer patients with prior cancer [1]. However, this trend has yet to be explored in Japan. The aim of this study was to provide globally comparable estimates of the prevalence, characteristics, and trends of prior cancer in newly diagnosed cancer patients using a long-term Japanese cancer registry database in order to aid further research and inform healthcare strategies.

## Methods

### Data source and study design

Data were obtained from the Osaka Cancer Registry (OCR), a population-based cancer registry founded in 1962 for the purpose of registering and monitoring all malignant tumors and benign intracranial tumors throughout Osaka prefecture (the third largest metropolitan area in Japan). The OCR covers a population of 8.8 million people, and allows the identification of prior cancers in individual patients [14].

Data on all cancer patients diagnosed between 1989 and 2015 were extracted for analysis. The various cancers were identified using the corresponding International Classification of Diseases, Tenth Revision (ICD-10) codes. The data also included each patient’s age at diagnosis (hereinafter referred to as diagnostic age), sex, cancer detection method, cancer stage, treatment, month of diagnosis (diagnostic month), year of diagnosis (diagnostic year), inclusion in the registry through death certificate only, living status, and survival time.

We quantified the prevalence of prior cancer in patients who received new diagnoses of cancer of the following sites between 2004 and 2015: lung, stomach, colorectum, female breast (hereinafter referred to as breast), cervix uteri, and corpus uteri. These sites were selected as they are the target sites for cancer screening programs in Japan, and are associated with high incidence [15]. We then examined the occurrence and types of prior cancers within 15 years before the diagnostic month and year of each newly diagnosed cancer.

### Definitions and study subjects

Index cancers were defined as cancers that were newly diagnosed between 2004 and 2015 in patients who met the following criteria: 1) diagnostic age of 15–99 years, 2) pathologically diagnosed cancer for any of the target sites (lung, stomach, colorectum, breast, cervix uteri, and corpus uteri), and 3) survived for three months or more after diagnosis. In Osaka prefecture, the percentage of cases registered from death certificate only fell below 10% from 2004 onward [16]. Therefore, data from 2004 and later were used to identify the index cancer cases. As prior cancers were identified within the 15-year period before each index cancer, data were extracted from 1989 (i.e., 15 years before 2004) onward. Because this study was conducted to provide evidence for the increased inclusion of cancer survivors in clinical trials, we focused on subjects who had survived for three months or more after diagnosis (i.e., patients with relatively longer survival). Patients that were registered in the OCR through death certificate only were excluded from this study because these cases often lack important patient background information, such as diagnosis date and cancer stage.

Cases of multiple cancers are recorded in the OCR in accordance with the guidelines of the International Agency for Research on Cancer and the International Association of Cancer Registries [17]. However, multiple cancers of the same site in an individual patient were combined as the “most common prior cancer” to avoid including the incomplete integration of multiple prior cancers or metastatic cancers [5].

Prior cancers were defined as cancers diagnosed during the 15-year period before the index cancer diagnosis. This 15-year cutoff was set to standardize the retroactive observation period across patients of different ages at the time of index cancer diagnosis and to provide a margin three times that of the 5-year window used in many clinical trials [13, 18]. If two or more prior cancers had the same diagnostic month and year in a patient, the sequence of these cancers was randomly determined using a previously described method [6].

We identified the index and prior cancer sites using the following ICD-10 codes [19]: all sites (C00–C96.x), mouth and pharynx (C00–C14.x), esophagus (C15.x), stomach (C16.x), colon (C18.x), rectum (C19.x–C20.x), liver (C22.x), gallbladder and bile duct (C23.x–C24.x), pancreas (C25.x), larynx (C32.x), lung (C33–C34.x), melanoma of skin (C43.x), other skin (C44.x), mesothelioma (C45.x), breast (C50.x), uterus (C53.x–C55.x), cervix uteri (C53.x), corpus uteri (C54.x), ovary (C56.x), prostate (C61.x), bladder (C67.x), renal and urinary tract (C64.x–C66.x, C68.x), brain and central nervous system (C70.x–C72.x), thyroid (C73.x), Hodgkin lymphoma (C81.x), non-Hodgkin lymphoma (C82.x–C86.x, C96.x), immunoproliferative diseases (C88.x), myeloma (C90.x), lymphoid leukemia (C91.x), acute myeloid leukemia (C92.0), myeloid leukemia (C92.x–C94.x), and unspecified leukemia (C95.x).

### Patient and index cancer characteristics

For each patient, we analyzed age group (15–39, 40–44, 45–49, 50–54, 55–59, 60–64, 65–69, 70–74, 75–79, 80–84, and 85–99 years), sex (male or female), method of cancer detection (screening and medical check-up, incidental detection during follow-up examination for another disease, and other or unknown; the last category generally involved cancer detection due to the occurrence of subjective symptoms) [20], cancer stage (localized, regional lymph nodes, regional extension, distant metastasis, and other or unknown), treatment (radiotherapy only, chemotherapy only, chemoradiotherapy, surgery only, surgery plus chemotherapy or radiotherapy, and other or unknown), and diagnostic year (2004–2005, 2006–2007, 2008–2009, 2010–2011, 2012–2013, or 2014–2015). Missing values were included in the “unknown” category for each factor.

### Prior cancer characteristics

For patients with prior cancer, we calculated the number of prior cancers before the index cancer, as well as the diagnostic time interval between the index cancer and most recent prior cancer. In addition, we examined the stage, treatment, and site of each prior cancer. The prior cancer site was identified for the most recent prior cancer. We categorized the following prior cancers as smoking-related cancers based on previous studies [21–25]: mouth, pharynx, larynx, lung, esophagus, stomach, liver, pancreas, kidney, urinary bladder, colorectum, cervix, and acute myeloid leukemia.

### Statistical analysis

The main outcome measure was the prevalence of prior cancer in the study subjects. In order to account for the varying age structures of the cancer patient population over time, we examined the trends in the age-adjusted prevalence (measured every two years) of prior cancer for each index cancer site according to sex. First, we calculated the age-specific prior cancer prevalence for each age group according to sex and cancer site. To obtain the expected number of prior cancer cases, we multiplied the age-specific prior cancer prevalence by the number of patients for each age group according to sex in our subjects between 2004 and 2015 (which was set as the reference cancer population). We then totaled the expected number of prior cancer cases from all age groups. Finally, to calculate the age-adjusted prevalence, we divided the total expected number of prior cancer cases by the reference cancer population. We described the temporal trends in prior cancer prevalence from 2004 to 2015 using the Cochran–Armitage trend test [26]. The distributions of the above site-specific measurements were also examined according to sex. The temporal trends in the method of index cancer detection were assessed.

Continuous variables were summarized as median values and interquartile ranges, and categorical variables were summarized as proportions. Proportions were compared using Pearson’s chi-square test. The significance level was set at 5% (two-sided). All analyses were performed using STATA version 14 (Stata corporation, College Station, TX, USA).

## Results

### Prior cancer prevalence and patient characteristics

**Fig. 1:**
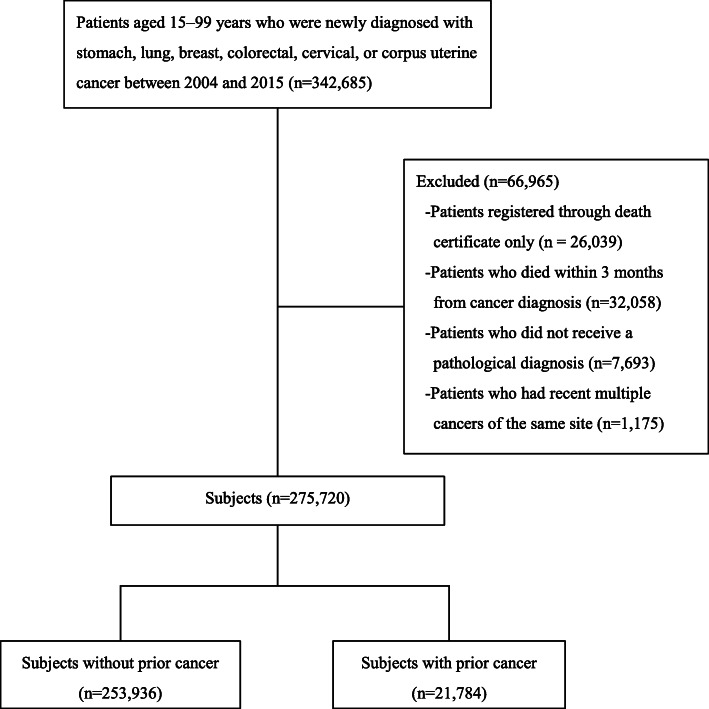
Flow diagram of study subject selection

**Fig. 2:**
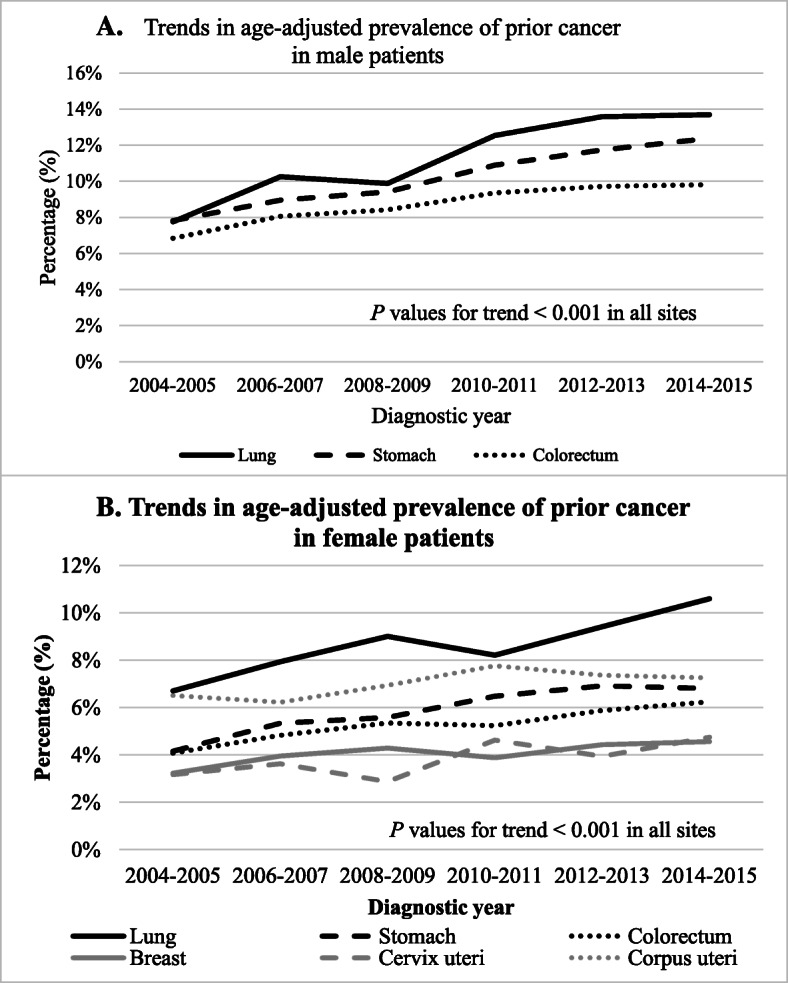
Trends in the age-adjusted prevalence of prior cancer between 2004 and 2015. The graphs show the age-adjusted prevalence of prior cancer in **a** male and **b** female patients according to index cancer site. *P* values for trend over the study period were calculated using the Cochran–Armitage test. The percentage of death certificate notification cases (DCN%) according to each diagnostic year was 26.2% in 2004–2005, 19.7% in 2006–2007, 16.5% in 2008–2009, 12.3% in 2010–2011, 9.0% in 2012–2013, and 6.0% in 2014–2015

Figure 1 shows the subject selection process. We identified 275,720 index cancer patients that met the inclusion and exclusion criteria. Among these, 21,784 (7.9%) had prior cancer. As shown in Fig. 2, the age-adjusted prevalence of prior cancer had significantly increased over the study period for all index cancers in both male and female patients (all *P* values < 0.001). The trends in age-adjusted index cancer prevalence and the expected number of index cancer patients with prior cancer are presented in Supplementary Table S1. This prevalence was notably higher in lung cancer patients in both sexes.

The characteristics of the patients according to index cancer site are summarized in Table 1. Among all patients, the prevalence of prior cancer was 11.1% in lung cancer patients, 9.5% in stomach cancer patients, 7.5% in colorectal cancer patients, 3.3% in breast cancer patients, 2.7% in cervical cancer patients, and 6.7% in corpus uterine cancer patients. Among older patients aged 65 years or older, the prevalence of prior cancer was 12.1% in lung cancer patients, 10.5% in stomach cancer patients, 8.4% in colorectal cancer patients, 5.2% in breast cancer patients, 5.4% in cervical cancer patients, and 8.5% in corpus uterine cancer patients. Throughout the study period, the proportion of cases detected through screening and medical check-up remained relatively stable, whereas the proportion of cases detected through incidental detection had increased (Supplementary Table S2). Patients whose index cancers were incidentally detected during follow-up examination for another disease had a higher prevalence of prior cancer than other cancer detection methods for all index cancer sites. In addition, the prevalence of prior cancer was also higher in patients whose index cancer was in the localized stage. With the exception of other or unknown treatments, surgery was the most common treatment for lung cancer (16.9%), colorectal cancer (8.2%), breast cancer (4.6%), and cervical cancer (5.2%).

**Table 1:** Characteristics of newly diagnosed cancer patients according to index cancer site

Index Cancer Site	Lung			Stomach			Colorectum			Breast			Cervix uteri			Corpus uteri		
	Total	Prior^a^		Total	Prior^a^		Total	Prior^a^		Total	Prior^a^		Total	Prior^a^		Total	Prior^a^	
*N* = 275,720	*N*	*N*	%	*N*	*N*	%	*N*	*N*	%	*N*	*N*	%	*N*	*N*	%	*N*	*N*	%
Total	56,930	6309	11.1	78,092	7382	9.5	77,032	5757	7.5	47,860	1588	3.3	7686	206	2.7	8120	542	6.7
Sex																		
Male	39,040	4628	11.9	54,479	5800	10.6	44,535	3841	8.6									
Female	17,890	1681	9.4	23,613	1582	6.7	32,497	1916	5.9	47,860	1588	3.3	7686	206	2.7	8120	542	6.7
Age, years																		
Median (IQR)	70 (63–76)	73 (67–78)		71 (63–77)	74 (68–79)		70 (62–77)	73 (67–79)		60 (48–69)	69 (61–76)		52 (41–66)	67 (56–77)		59 (51–68)	62 (52–71)	
15–39	455	15	3.3	864	8	0.9	961	10	1.0	3034	23	0.8	1706	10	1.2	437	18	4.1
40–44	551	20	3.6	919	17	1.8	1191	29	2.4	4556	35	0.8	998	8	2.1	447	25	5.6
45–49	1032	39	3.8	1572	39	2.5	1974	47	2.4	5737	61	1.1	806	15	4.2	761	57	7.5
50–54	1965	98	5.0	2954	107	3.6	3508	103	2.9	4885	91	1.9	650	13	5.4	1121	87	7.8
55–59	4197	242	5.8	5962	292	4.9	6471	277	4.3	5589	137	2.5	712	21	3.5	1375	59	4.3
60–64	7902	671	8.5	10,240	658	6.4	10,754	559	5.2	6376	214	3.4	733	26	4.3	1261	64	5.1
65–69	10,790	1110	10.3	13,402	1197	8.9	13,329	936	7.0	5889	279	4.7	635	19	5.1	1000	67	6.7
70–74	12,028	1419	11.8	15,508	1686	10.9	14,275	1259	8.8	4693	277	5.9	550	27	7.3	753	68	9.0
75–79	10,191	1503	14.7	13,493	1774	13.1	12,180	1255	10.3	3429	222	6.5	396	29	7.5	534	42	7.9
80–84	5702	875	15.3	8550	1077	12.6	7568	857	11.3	2160	153	7.1	271	19	10.5	298	42	14.1
85–99	2117	317	15.0	4628	527	11.4	4821	425	8.8	1512	96	6.3	229	19	7.8	133	13	9.8
Method of cancer detection																		
Screening and medical check-up	8417	399	4.7	11,938	553	4.6	11,299	466	4.1	9565	167	1.7	1580	27	3.3	702	50	7.1
Incidental detection^b^	19,365	4222	21.8	20,721	4003	19.3	15,244	2728	17.9	3938	666	16.9	742	56	11.7	1173	180	15.3
Other or unknown^c^	29,148	1688	5.8	45,433	2826	6.2	50,489	2563	5.1	34,357	755	2.2	5364	123	3.6	6245	312	5.0
Cancer stage																		
Localized	17,540	2986	17.0	43,352	5027	11.6	35,289	3152	8.9	28,498	1087	3.8	3566	90	5.0	5314	373	7.0
Regional lymph nodes	7112	785	11.0	9327	725	7.8	15,269	1036	6.8	11,856	343	2.9	382	6	3.2	266	15	5.6
Regional extension	8188	782	9.6	8477	509	6.0	9034	581	6.4	2179	44	2.0	2529	80	3.8	1577	85	5.4
Distant metastasis	20,480	1388	6.8	12,500	688	5.5	12,838	659	5.1	2340	48	2.1	666	12	3.8	587	37	6.3
Other or unknown	3610	368	10.2	4436	433	9.8	4602	329	7.1	2987	66	2.2	543	18	5.1	376	32	8.5
Treatment																		
Radiotherapy only	3116	471	15.1	48	14	29.2	93	6	6.5	214	3	1.4	823	38	4.9	67	6	9.0
Chemotherapy only	15,195	1076	7.1	7455	543	7.3	1974	142	7.2	1984	38	1.9	224	4	4.0	175	13	7.4
Chemoradiotherapy	7394	515	7.0	166	38	22.9	184	13	7.1	185	4	2.2	1177	32	2.9	21	3	14.3
Surgery only	15,772	2665	16.9	29,975	2514	8.4	40,687	3331	8.2	16,277	741	4.6	2645	64	5.2	4059	298	7.3
Surgery plus chemotherapy or radiotherapy	6839	634	9.3	13,618	772	5.7	23,206	1176	5.1	24,665	639	2.6	2057	37	3.7	3277	178	5.4
Other or unknown	8614	948	11.0	26,830	3501	13.0	10,888	1089	10.0	4535	163	3.6	760	31	5.7	521	44	8.4
Diagnostic year																		
2004–2005	7288	538	7.4	9969	635	6.4	9151	498	5.4	5650	143	2.5	1025	23	4.2	876	61	7.0
2006–2007	8407	803	9.6	11,542	892	7.7	11,295	743	6.6	6913	206	3.0	1098	26	3.8	1029	60	5.8
2008–2009	8880	856	9.6	12,498	1048	8.4	11,833	825	7.0	7297	244	3.3	1174	28	4.1	1136	75	6.6
2010–2011	10,240	1195	11.7	13,806	1373	9.9	13,565	1052	7.8	8596	270	3.1	1416	39	4.7	1542	104	6.7
2012–2013	10,641	1379	13.0	15,047	1661	11.0	15,074	1252	8.3	9050	337	3.7	1451	39	4.8	1720	122	7.1
2014–2015	11,474	1538	13.4	15,230	1773	11.6	16,114	1387	8.6	10,354	388	3.7	1522	51	4.9	1817	120	6.6

*Abbreviation*: *IQR* interquartile range

Categorical variables between cancer patients with and without prior cancer were compared using Pearson’s chi-square test. Significant differences (*P* ≤ 0.05) were found in all categories except for diagnostic year in corpus uterine cancer patients. ^a^Newly diagnosed cancer patients with prior cancer of the specified site. ^b^Detected during follow-up examination for another disease. ^c^Generally detected from the occurrence of subjective symptoms

### Prior cancer characteristics

The characteristics of male and female patients with prior cancer are shown in Tables 2 and 3, respectively. Approximately 90% of these patients had only one prior cancer regardless of sex. The proportions of male patients with two prior cancers were 10.4% for lung cancer, 8.7% for stomach cancer, and 8.9% for colorectal cancer. In female patients, these proportions were 7.2% for lung cancer, 5.6% for stomach cancer, 6.1% for colorectal cancer, 5.0% for breast cancer, 7.3% for cervical cancer, and 5.4% for corpus uterine cancer. The cumulative proportions of the most recent prior cancers diagnosed within 5 years before the index cancer diagnosis were 69.4% (male) and 65.0% (female) for lung cancer, 75.0% (male) and 67.4% (female) for stomach cancer, 75.5% (male) and 65.3% (female) for colorectal cancer, 65.2% (female) for breast cancer, 69.0% (female) for cervical cancer, and 54.7% (female) for corpus uterine cancer. The most common and least common prior cancer stages were localized and distant metastasis, respectively. Surgery only was the most common treatment for prior cancer across all index cancer sites in both sexes.

**Table 2:** Characteristics of newly diagnosed male cancer patients with prior cancer

Index Cancer Site	Lung		Stomach		Colorectum	
	*N*	%	*N*	%	*N*	%
Total	4628	100	5800	100	3841	100
Number of prior cancers before the index cancer						
1	4100	88.6	5256	90.6	3457	90.0
2	481	10.4	507	8.7	343	8.9
3	44	1.0	35	0.6	39	1.0
4	2	0.0	2	0.0	1	0.0
5	1	0.0	0	0.0	1	0.0
Diagnostic time interval^a^						
< 3 months	741	16.0	1687	29.1	1240	32.3
3 months–1 year	555	12.0	656	11.3	428	11.1
1–5 years	1916	41.4	2006	34.6	1232	32.1
5–10 years	988	21.3	1017	17.5	641	16.7
10–15 years	428	9.2	434	7.5	300	7.8
Stage of prior cancer^b^						
Localized	2941	63.5	3320	57.2	2308	60.1
Regional lymph nodes	455	9.8	650	11.2	350	9.1
Regional extension	528	11.4	797	13.7	501	13.0
Distant metastasis	229	4.9	453	7.8	325	8.5
Other or unknown	475	10.3	580	10.0	357	9.3
Treatment of prior cancer^b^						
Radiotherapy only	185	4.0	265	4.6	152	4.0
Chemotherapy only	252	5.4	438	7.6	310	8.1
Chemoradiotherapy	148	3.2	296	5.1	141	3.7
Surgery only	1986	42.9	2246	38.7	1468	38.2
Surgery plus chemotherapy or radiotherapy	631	13.6	893	15.4	535	13.9
Other or unknown	1426	30.8	1662	28.7	1235	32.2
Site of prior cancer^b^						
Mouth and pharynx	209	4.5	254	4.4	137	3.6
Esophagus	236	5.1	646	11.1	172	4.5
Stomach	1290	27.9	–	–	1302	33.9
Colon	543	11.7	970	16.7	–	–
Rectum	342	7.4	602	10.4	–	–
Liver	234	5.1	570	9.8	278	7.2
Gallbladder and bile duct	40	0.9	75	1.3	56	1.5
Pancreas	45	1.0	69	1.2	35	0.9
Larynx	172	3.7	198	3.4	90	2.3
Lung	–	–	591	10.2	386	10.0
Melanoma of skin	4	0.1	7	0.1	4	0.1
Other skin	73	1.6	67	1.2	66	1.7
Mesothelioma	4	0.1	7	0.1	9	0.2
Prostate	687	14.8	924	15.9	646	16.8
Bladder	265	5.7	281	4.8	213	5.5
Renal and urinary tract	164	3.5	185	3.2	172	4.5
Brain and central nervous system	5	0.1	8	0.1	7	0.2
Thyroid	38	0.8	42	0.7	38	1.0
Hodgkin lymphoma	12	0.3	4	0.1	8	0.2
Non-Hodgkin lymphoma	118	2.5	142	2.4	97	2.5
Immunoproliferative diseases	1	0.0	1	0.0	0	0.0
Myeloma	18	0.4	19	0.3	17	0.4
Lymphoid leukemia	13	0.3	11	0.2	8	0.2
Myeloid leukemia	13	0.3	17	0.3	14	0.4
Leukemia unspecified	0	0.0	0	0.0	1	0.0
Others	102	2.2	110	1.9	85	2.2

Values are expressed as the number of patients and column percentage

^a^Interval between the diagnostic dates of the most recent prior cancer and index cancer

^b^Most recently diagnosed prior cancer

**Table 3:** Characteristics of newly diagnosed female cancer patients with prior cancer

Index Cancer Site	Lung		Stomach		Colorectum		Breast		Cervix uteri		Corpus uteri	
	*N*	%	*N*	%	*N*	%	*N*	%	*N*	%	*N*	%
Total	1681	100	1582	100	1916	100	1588	100	206	100	542	100
Number of prior cancers before the index cancer												
1	1551	92.3	1488	94.1	1794	93.6	1507	94.9	191	92.7	510	94.1
2	121	7.2	88	5.6	116	6.1	79	5.0	15	7.3	29	5.4
3	9	0.5	6	0.4	5	0.3	2	0.1	0	0.0	3	0.6
4	0	0.0	0	0.0	1	0.1	0	0.0	0	0.0	0	0.0
Diagnostic time interval^a^												
< 3 months	282	16.8	374	23.6	445	23.2	317	20.0	43	20.9	107	19.7
3 months–1 year	188	11.2	151	9.5	173	9.0	177	11.1	28	13.6	38	7.0
1–5 years	623	37.1	541	34.2	634	33.1	548	34.5	71	34.5	152	28.0
5–10 years	373	22.2	324	20.5	421	22.0	357	22.5	40	19.4	156	28.8
10–15 years	215	12.8	192	12.1	243	12.7	189	11.9	24	11.7	89	16.4
Stage of prior cancer^b^												
Localized	979	58.2	917	58.0	1155	60.3	882	55.5	105	51.0	300	55.4
Regional lymph nodes	285	17.0	233	14.7	275	14.4	232	14.6	40	19.4	104	19.2
Regional extension	185	11.0	174	11.0	202	10.5	212	13.4	15	7.3	66	12.2
Distant metastasis	66	3.9	100	6.3	117	6.1	112	7.1	13	6.3	20	3.7
Other or unknown	166	9.9	158	10.0	167	8.7	150	9.4	33	16.0	52	9.6
Treatment of prior cancer^b^												
Radiotherapy only	24	1.4	32	2.0	37	1.9	28	1.8	3	1.5	10	1.8
Chemotherapy only	65	3.9	97	6.1	123	6.4	88	5.5	13	6.3	25	4.6
Chemoradiotherapy	41	2.4	28	1.8	27	1.4	31	2.0	4	1.9	2	0.4
Surgery only	784	46.6	747	47.2	860	44.9	848	53.4	89	43.2	217	40.0
Surgery plus chemotherapy or radiotherapy	484	28.8	403	25.5	518	27.0	368	23.2	50	24.3	215	39.7
Other or unknown	283	16.8	275	17.4	351	18.3	225	14.2	47	22.8	73	13.5
Site of prior cancer^b^												
Mouth and pharynx	50	3.0	32	2.0	29	1.5	39	2.5	4	1.9	8	1.5
Esophagus	24	1.4	62	3.9	26	1.4	27	1.7	2	1.0	1	0.2
Stomach	239	14.2	–	–	445	23.2	250	15.7	31	15.0	29	5.4
Colon	203	12.1	320	20.2	–	–	252	15.9	22	10.7	46	8.5
Rectum	95	5.7	132	8.3	–	–	133	8.4	13	6.3	19	3.5
Liver	48	2.9	104	6.6	93	4.9	58	3.7	9	4.4	3	0.6
Gallbladder and bile duct	14	0.8	36	2.3	35	1.8	13	0.8	2	1.0	6	1.1
Pancreas	30	1.8	31	2.0	28	1.5	13	0.8	0	0.0	4	0.7
Larynx	13	0.8	2	0.1	4	0.2	2	0.1	3	1.5	0	0
Lung	0	0.0	96	6.1	151	7.9	155	9.8	12	5.8	25	4.6
Melanoma of skin	1	0.1	3	0.2	3	0.2	3	0.2	0	0.0	1	0.2
Other skin	27	1.6	42	2.7	39	2.0	30	1.9	3	1.5	12	2.2
Mesothelioma	1	0.1	0	0.0	0	0.0	0	0.0	0	0.0	0	0.0
Breast	485	28.9	378	23.9	528	27.6	–	–	54	26.2	253	46.7
Cervix uteri	72	4.3	61	3.9	62	3.2	100	6.3	0	0.0	23	4.2
Corpus uteri	66	3.9	45	2.8	108	5.6	132	8.3	12	5.8	0	0.0
Uterus	6	0.4	3	0.2	5	0.3	7	0.4	2	1.0	1	0.2
Ovary	46	2.7	25	1.6	56	2.9	60	3.8	4	1.9	44	8.1
Bladder	27	1.6	27	1.7	38	2.0	23	1.4	4	1.9	10	1.8
Renal and urinary tract	49	2.9	26	1.6	67	3.5	51	3.2	7	3.4	12	2.2
Brain and central nervous system	6	0.4	4	0.3	3	0.2	1	0.1	0	0.0	3	0.6
Thyroid	86	5.1	40	2.5	68	3.5	131	8.2	1	0.5	17	3.1
Hodgkin lymphoma	4	0.2	1	0.1	3	0.2	1	0.1	1	0.5	0	0.0
Non-Hodgkin lymphoma	38	2.3	56	3.5	50	2.6	48	3.0	8	3.9	15	2.8
Immunoproliferative diseases	0	0.0	0	0.0	1	0.1	1	0.1	0	0.0	0	0.0
Myeloma	2	0.1	9	0.6	12	0.6	7	0.4	2	1.0	2	0.4
Lymphoid leukemia	4	0.2	2	0.1	2	0.1	1	0.1	2	1.0	0	0.0
Myeloid leukemia	4	0.2	7	0.4	8	0.4	9	0.6	1	0.5	2	0.4
Others	41	2.4	38	2.4	52	2.7	41	2.6	7	3.4	6	1.1

Values are expressed as the number of patients and column percentage

^a^Interval between the diagnostic dates of the most recent prior cancer and index cancer

^b^Most recently diagnosed prior cancer

**Fig. 3:**
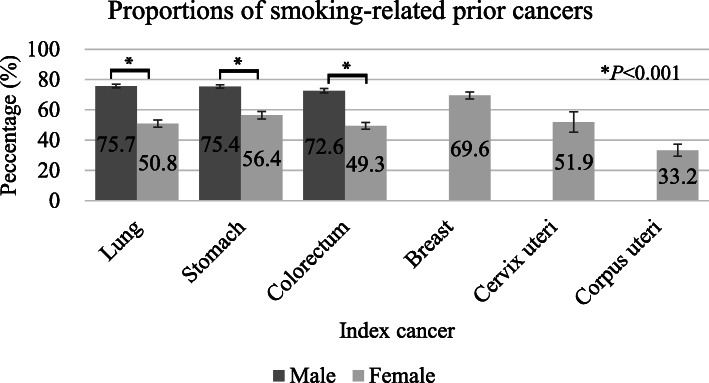
Proportions of smoking-related prior cancers according to index cancer site. Smoking-related prior cancers included cancers of the mouth, pharynx, larynx, lung, esophagus, stomach, liver, pancreas, kidney, urinary bladder, colorectum, cervix, and acute myeloid leukemia. Pearson’s chi-square test was used to compare the proportions of smoking-related prior cancers between the sexes. Error bars represent 95% confidence intervals

The most common sites of the most recent prior cancers were analyzed according to index cancer site in male patients (Table 2) and female patients (Table 3). In male patients, smoking-related cancers accounted for approximately 70% of prior cancers. In female patients, smoking-related prior cancers were more common in index breast cancer patients and less common in index corpus uterine cancer patients than patients with the other index cancers (Fig. 3). The proportions of smoking-related prior cancers in male patients were significantly higher than in female patients for index lung, stomach, and colorectal cancer (Fig. 3). Supplementary Table S3 presents the temporal trends in the proportion of smoking-related index cancers, and Supplementary Table S4 summarizes the temporal trends in the proportion of smoking-related prior cancers among index cancer patients.

## Discussion

In this analysis of population-based cancer registry data from a major metropolitan area in Japan, we ascertained the prevalence, characteristics, and trends of prior cancer in patients newly diagnosed with one of five major cancer types. Even after accounting for the changes in age structure, the prevalence of prior cancer was found to have increased over time for all index cancer sites. These trends may be attributable to earlier cancer detection (increase in localized cancer rates from 35.2% in 2004 to 48.4% in 2015). The screening and medical check-up method can result in earlier detection, but the use of this method was relatively stable throughout the study period. In contrast, cancers detected using the incidental detection method (where cancer is detected during follow-up examination for another disease) had increased over the study period. This may have contributed to the earlier detection of cancers, which subsequently led to the rising prevalence of prior cancer in recent years. The increasing trends in the prevalence of prior cancer may also be attributable to longer survival (increase in relative survival in all cancers from 46.6% in 2004 to 59.2% in 2010) and expanded cancer registry coverage (decrease in the percentages of cases with death certificate notification or death certificate only) in more recently diagnosed cancer patients [15, 27, 28].

Among our subjects, patients with newly diagnosed lung cancer had the highest prevalence of prior cancer among the assessed index cancers. These differences in the prevalences of prior cancer may be indicative of underlying or shared risk factors (e.g., lifestyle habits such as smoking), and require further investigation.

Our analysis showed that the prevalence of prior cancer increased with age for all index cancers, with adolescents and young adults (aged 15–39 years) having a lower prevalence than older patients. However, our estimated prevalences of prior cancer in patients aged 65 years or older were lower than those reported in a previous study conducted in the US (lung: 18.7%, stomach: 17.8%, colorectum: 15.3%, breast: 7.4%, cervix: 13.6%, and corpus uteri: 13.6%) [6]. This disparity may be influenced by an inherent difference in the age-standardized cancer incidence rates for all sites between the US (393.2 per 100,000 population) and Japan (285.9 per 100,000 population) [29]. In addition to variations in prior cancer prevalence, this may also be indicative of differences in genetic, lifestyle-related, and/or environmental risk factors. Differences in the prevalence of prior cancers among previous studies may also be explained in part by our non-inclusion of patients with carcinoma in situ, as this condition is generally curable and would not unduly affect survival. In addition, we did not use the recorded sequence numbers that indicate the order of cancer in individual cases, which may also have resulted in a lower apparent prevalence of prior cancer. We had decided to exclude prior cancers at the same site as the index cancer to avoid potential double counting because we would be unable to determine if the index cancer was metastatic or primary.

The prevalence of prior cancer was found to be higher in index cancer patients with localized tumors, and prior cancers were generally diagnosed in the early localized stage across all index cancers. A possible explanation for the former observation is that patients with prior cancer would undergo regular follow-up examinations, which would support the prompt incidental detection of new tumors in the early stages. This may also contribute to the high prevalence of prior cancer for patients whose index cancers were incidentally detected during examinations for another disease. For the latter observation, we posit that patients with cancers diagnosed in the earlier stages would receive prompt treatment and have longer survival, thereby increasing the opportunities for other cancers to develop. Our results also showed that surgical treatment was often selected for prior cancer, which may be due to the high proportion of early-stage cancers.

In the present study, the prevalence of prior cancers in male patients was higher than in female patients. In particular, the proportion of male patients with two or more prior cancers was higher than female patients for new cases of lung cancer, stomach cancer, and colorectal cancer. The cumulative proportions of the most recent prior cancers diagnosed within 5 years before the index cancer diagnosis were higher in male patients (Tables 2 and 3). These observations may be influenced by the higher incidence of cancers in men, lifestyle differences, and other sex-based differences [30].

While our data did not include information on tobacco consumption, smoking-related prior cancers were found to be less common in female patients newly diagnosed with lung, stomach, and colorectal cancer than their male counterparts. The proportions of smoking-related prior cancers were 73.1% in male patients and 53.0% in female patients among the general population of cancer patients (*P* < 0.001). In recent years, smoking rates have declined in Japan (45.7% in men and 15.2% in women in 2004 to 30.4% in men and 10.7% in women in 2016) [31]. At the same time, smoking-related cancers have also decreased, suggesting that the reduction in smokers is related to the reduction in smoking-related cancers (Supplementary Table S3). However, the proportion of index cancer patients with smoking-related prior cancers have decreased in men, but increased in women (Supplementary Table S4). Smoking prevalence may be associated with smoking-related prior cancers among male patients, but this relationship is less clear in female patients. This may indicate a biological difference in susceptibility to smoking-related cancer between the sexes, but more work is needed to explore this possibility [32, 33].

Furthermore, female patients with index breast cancer had a higher proportion of smoking-related prior cancers than those with other index cancers. Although several studies have reported that smoking is associated with an increased risk of breast cancer [34–36], the evidence remains inconclusive and further research is needed to understand these findings. Patients with cervical cancer had a significantly higher rate of smoking-related cancers than those with corpus uterine cancer, likely because cervical cancer is also a smoking-related cancer.

Our results also showed that patients with index breast cancer had a higher prevalence of prior corpus uterine cancer (8.3%) relative to the general cancer population (3.7%) in the OCR [15]. Similarly, patients with index corpus uterine cancer had a higher prevalence of prior breast cancer (46.6%) relative to the general cancer population (20.7%) in the OCR [15]. In addition, there was a slightly higher prevalence of prior ovary cancer in patients with index breast cancer (3.8%) than the general cancer population (2.4%) [15]. This may be indicative of hereditary cancers (such as hereditary breast and ovarian cancer syndrome, Lynch syndrome, and Li-Fraumeni syndrome) or the effects of previous treatments for prior cancers [37, 38].

Cases with prior cancer are frequently excluded from clinical research. However, the relatively high prevalence of prior cancer in newly diagnosed cancer patients suggests that their exclusion would have a substantial effect on research outcomes. Approximately 80% of previous clinical trials for lung cancer patients excluded those with prior cancer, and most trials employ a 5-year exclusion window [13, 18]. Patients with prior cancer are also sometimes excluded from observational studies due to concerns that they may affect outcome measurements [39, 40]. Our present study found that approximately 70% of lung, stomach, and colorectal cancer patients with prior cancer had a diagnostic interval of 5 years or less between the prior and index cancers. In addition, the most frequent diagnostic interval was 1–5 years for all index cancer sites. Even after excluding cancer patients with a diagnostic interval of 3 months or less, individuals who had a diagnostic interval of 5 years or less still accounted for more than 60% of patients with prior cancer. Accordingly, a considerable proportion of patients with prior cancer would not be eligible to participate in trials with a 5-year exclusion window. The impact of prior cancer on survival should be examined with scientific evidence, and cancer survivors should not be excluded from studies as a matter of course [41]. Quantifying the prevalence of prior cancer in newly diagnosed cancer patients can aid our understanding of these patients and support the formulation of comprehensive treatment strategies. For example, medical institutions, government agencies, and insurers may be able to design more efficient strategies to allocate health resources and develop treatment plans that account for cancer survivors [42–45].

This study has the following limitations. First, we created sequence numbers for multiple cancers in the OCR with the assumption that patients did not move outside of the cancer registry catchment area (i.e., Osaka prefecture). If the population had grown during the study period, the index cancer prevalences may increase, but the prior cancer prevalences could be underestimated due to a reduced coverage of medical history in new residents. If the population had declined during the study period, the index cancer prevalences may fall, and the accuracy of prior cancer prevalences may also be affected. In Osaka prefecture, out-migration exceeded in-migration until 2010, and this pattern was reversed from 2011 onward [46]. Overall, the prefectural population had increased from 8.81 million in 2004 to 8.83 million in 2015 [46]. Among the age groups, there were many in-migrants aged 15–24 years, whereas the other age groups tended to be out-migrants [46]. Accordingly, it is possible that the prevalences of index cancers and prior cancers were underestimated. Second, the registry lacked several types of patient information (e.g., tobacco use, alcohol use, and obesity), which prevented a more detailed analysis of patient background factors. Third, our study did not examine the competing risk of dying from the first cancer (which would preclude the occurrence of a second cancer) when considering second cancers in patients. Furthermore, cancers diagnosed in the earlier stages may involve lead time bias or length time bias because of earlier detection and slowly progressing diseases. Future studies are therefore needed to explore these aspects. Fourth, it is possible that cancers that occurred at a younger age had been overlooked due to the use of a 15-year retroactive observation period for identifying prior cancers.

Despite these limitations, our study has several strengths. Using a historic, large-scale cancer registry database, we were able to identify and characterize prior cancers in newly diagnosed cancer patients residing in a major metropolitan area in Japan. To the best of our knowledge, this is the first study to reveal recent trends in the age-adjusted prevalence of prior cancers stratified by sex.

## Conclusions

This study provided globally comparable estimates of the prevalence and characteristics of prior cancer in newly diagnosed cancer patients using a long-term population-based cancer registry. The prevalence of prior cancer increased in recent years, and approximately 70% of prior cancers in male patients were potentially smoking-related. In light of the increasing number of cancer survivors worldwide, our findings suggest that a deeper understanding of the prevalence and characteristics of prior cancer in cancer patients is needed to promote more inclusive clinical research and support the expansion of treatment options.

## Supplementary Information

**Additional file 1: Supplementary Table S1.** Temporal trends in age-adjusted index cancer prevalence and the expected number of index cancer patients with prior cancer

**Additional file 2: Supplementary Table S2.** Temporal trends in the method of index cancer detection

**Additional file 3: Supplementary Table S3.** Temporal trends in the proportion of smoking-related index cancers

**Additional file 4: Supplementary Table S4.** Temporal trends in the proportion of smoking-related prior cancers among index cancer patients

## Data Availability

The datasets used and/or analyzed during the current study are available from the corresponding author on reasonable request.

## Abbreviations

OCR: Osaka Cancer Registry
ICD-10: International Classification of Diseases, Tenth Revision
