# Supplementary material for: Increasing trends in the prevalence of prior cancer in newly diagnosed lung, stomach, colorectal, breast, cervical, and corpus uterine cancer patients: a population-based study
Source: BMC Cancer. 2021 Mar 10;21:264. doi: 10.1186/s12885-021-08011-3 (PMC7948331; doi:10.1186/s12885-021-08011-3)
Supplement: Supplementary file 1 — Additional file 1: Supplementary Table S1. Temporal trends in age-adjusted index cancer prevalence and the expected number of index cancer patients with prior cancer [file 12885_2021_8011_MOESM1_ESM.docx]

**Additional File 1**

**Supplementary Table S1.** **Temporal trends in age-adjusted index cancer prevalence and the expected number of** **index cancer patients with prior cancer**

|  |  |  | | | | | | | | | | | |
| --- | --- | --- | --- | --- | --- | --- | --- | --- | --- | --- | --- | --- | --- |
| Diagnostic year | Index Cancer Site | 2004–2005 | | 2006–2007 | | 2008–2009 | | 2010–2011 | | 2012–2013 | | 2014–2015 | |
|  |  | % | Expected N | % | Expected N | % | Expected N | % | Expected N | % | Expected N | % | Expected N |
| Male | Lung | 7.8 | 10,704 | 10.2 | 14,151 | 9.9 | 13,636 | 12.5 | 17,302 | 13.6 | 18,751 | 13.7 | 18,893 |
|  | Stomach | 7.8 | 10,789 | 9.0 | 12,365 | 9.4 | 12,995 | 10.9 | 15,030 | 11.7 | 16,195 | 12.4 | 17,087 |
|  | Colorectum | 6.8 | 9,445 | 8.1 | 11,127 | 8.4 | 11,618 | 9.4 | 12,917 | 9.7 | 13,410 | 9.8 | 13,541 |
|  |  |  |  |  |  |  |  |  |  |  |  |  |  |
| Female | Lung | 6.7 | 9,239 | 7.9 | 10,932 | 9.0 | 12,415 | 8.2 | 11,322 | 9.4 | 12,966 | 10.6 | 14,608 |
|  | Stomach | 4.2 | 5,732 | 5.3 | 7,350 | 5.6 | 7,692 | 6.5 | 8,928 | 6.9 | 9,527 | 6.8 | 9,381 |
|  | Colorectum | 4.1 | 5,619 | 4.8 | 6,656 | 5.3 | 7,372 | 5.2 | 7,209 | 5.9 | 8,099 | 6.2 | 8,605 |
|  | Breast | 3.2 | 4,450 | 3.9 | 5,439 | 4.3 | 5,913 | 3.9 | 5,342 | 4.4 | 6,111 | 4.6 | 6,294 |
|  | Cervix uteri | 3.2 | 4,369 | 3.6 | 4,991 | 2.9 | 3,953 | 4.6 | 6,379 | 3.9 | 5,426 | 4.7 | 6,547 |
|  | Corpus uteri | 6.5 | 8,977 | 6.2 | 8,583 | 6.9 | 9,565 | 7.8 | 10,693 | 7.4 | 10,150 | 7.3 | 9,996 |
| To obtain the expected number of prior cancer cases, we multiplied the age-specific prior cancer prevalence by the number of cancer patients for each age group according to sex in our subjects between 2004 and 2015 (which was set as the reference cancer population). We then totaled the expected number of prior cancer cases from all age groups. | | | | | | | | | | | | | |
