# Supplementary material for: Increasing trends in the prevalence of prior cancer in newly diagnosed lung, stomach, colorectal, breast, cervical, and corpus uterine cancer patients: a population-based study
Source: BMC Cancer. 2021 Mar 10;21:264. doi: 10.1186/s12885-021-08011-3 (PMC7948331; doi:10.1186/s12885-021-08011-3)
Supplement: Supplementary file 2 — Additional file 2: Supplementary Table S2. Temporal trends in the method of index cancer detection [file 12885_2021_8011_MOESM2_ESM.docx]

**Additional File 2**

**Supplementary Table S2.** **Temporal trends in the method of index cancer detection**

| Diagnostic year | 2004–2005 | | | 2006–2007 | | | 2008–2009 | | | 2010–2011 | | | 2012–2013 | | | 2014–2015 | | |
| --- | --- | --- | --- | --- | --- | --- | --- | --- | --- | --- | --- | --- | --- | --- | --- | --- | --- | --- |
|  | No prior^a^ | Prior^b^ | Total | No prior^a^ | Prior^b^ | Total | No prior^a^ | Prior^b^ | Total | No prior^a^ | Prior^b^ | Total | No prior^a^ | Prior^b^ | Total | No prior^a^ | Prior^b^ | Total |
| Screening and medical check-up | 4,087 | 149 | 4,236 | 6,226 | 239 | 6,465 | 6,704 | 236 | 6,940 | 7,690 | 268 | 7,958 | 8,301 | 388 | 8,689 | 8,831 | 380 | 9,211 |
|  | 12.75 | 7.87 | 12.48 | 16.58 | 8.77 | 16.05 | 16.87 | 7.69 | 16.21 | 17.04 | 6.65 | 16.19 | 17.22 | 8.12 | 16.4 | 17.23 | 7.24 | 16.3 |
|  |  |  |  |  |  |  |  |  |  |  |  |  |  |  |  |  |  |  |
| Incidental detection^c^ | 4,099 | 687 | 4,786 | 6,192 | 1,285 | 7,477 | 7,732 | 1,617 | 9,349 | 8,917 | 2,237 | 11,154 | 10,701 | 2,803 | 13,504 | 11,687 | 3,209 | 14,896 |
|  | 12.79 | 36.27 | 14.1 | 16.49 | 47.17 | 18.56 | 19.46 | 52.69 | 21.84 | 19.76 | 55.54 | 22.69 | 22.2 | 58.63 | 25.49 | 22.8 | 61.12 | 26.36 |
|  |  |  |  |  |  |  |  |  |  |  |  |  |  |  |  |  |  |  |
| Other or unknown^d^ | 23,875 | 1,058 | 24,933 | 25,136 | 1,200 | 26,336 | 25,306 | 1,216 | 26,522 | 28,525 | 1,523 | 30,048 | 29,191 | 1,590 | 30,781 | 30,736 | 1,661 | 32,397 |
|  | 74.47 | 55.86 | 73.43 | 66.93 | 44.05 | 65.39 | 63.68 | 39.62 | 61.95 | 63.2 | 37.81 | 61.12 | 60.57 | 33.26 | 58.11 | 59.97 | 31.64 | 57.34 |
| ^a^Newly diagnosed cancer patients with no prior cancer. ^b^Newly diagnosed cancer patients with prior cancer. ^c^Detected during follow-up examination for another disease. ^d^Generally detected from the occurrence of subjective symptoms. | | | | | | | | | | | | | | | | | | |
