# Supplementary material for: Increasing trends in the prevalence of prior cancer in newly diagnosed lung, stomach, colorectal, breast, cervical, and corpus uterine cancer patients: a population-based study
Source: BMC Cancer. 2021 Mar 10;21:264. doi: 10.1186/s12885-021-08011-3 (PMC7948331; doi:10.1186/s12885-021-08011-3)
Supplement: Supplementary file 3 — Additional file 3: Supplementary Table S3. Temporal trends in the proportion of smoking-related index cancers [file 12885_2021_8011_MOESM3_ESM.docx]

**Additional File 3**

**Supplementary Table S3. Temporal trends in the proportion of smoking-related index cancers**

|  | | | | | | | | | | | | |
| --- | --- | --- | --- | --- | --- | --- | --- | --- | --- | --- | --- | --- |
| Diagnostic year | 2004–2005 | | 2006–2007 | | 2008–2009 | | 2010–2011 | | 2012–2013 | | 2014–2015 | |
|  | N | % | N | % | N | % | N | % | N | % | N | % |
| Smoking-related cancers |  |  |  |  |  |  |  |  |  |  |  |  |
| Male | 37,690 | 80.1 | 42,423 | 78.3 | 44,642 | 76.7 | 48,173 | 74.3 | 51,452 | 73.5 | 53,568 | 72.8 |
| Female | 19,878 | 60.0 | 22,744 | 58.7 | 23,734 | 58.3 | 26,447 | 57.5 | 28,137 | 56.9 | 29,781 | 55.8 |
| Total | 57,568 | 71.8 | 65,167 | 70.1 | 68,376 | 69.1 | 74,620 | 67.3 | 79,589 | 66.6 | 83,349 | 65.6 |
|  |  |  |  |  |  |  |  |  |  |  |  |  |
| Non-smoking-related cancers |  |  |  |  |  |  |  |  |  |  |  |  |
| Male | 9,340 | 19.9 | 11,781 | 21.7 | 13,602 | 23.4 | 16,695 | 25.7 | 18,537 | 26.5 | 20,006 | 27.2 |
| Female | 13,234 | 40.0 | 16,028 | 41.3 | 16,972 | 41.7 | 19,588 | 42.6 | 21,335 | 43.1 | 23,631 | 44.2 |
| Total | 22,574 | 28.2 | 27,809 | 29.9 | 30,574 | 30.9 | 36,283 | 32.7 | 39,872 | 33.4 | 43,637 | 34.4 |
| Total |  |  |  |  |  |  |  |  |  |  |  |  |
| Male | 47,030 | 100 | 54,204 | 100 | 58,244 | 100 | 64,868 | 100 | 69,989 | 100 | 73,574 | 100 |
| Female | 33,112 | 100 | 38,772 | 100 | 40,706 | 100 | 46,035 | 100 | 49,472 | 100 | 53,412 | 100 |
| Total | 80,142 | 100 | 92,976 | 100 | 98,950 | 100 | 110,903 | 100 | 119,461 | 100 | 126,986 | 100 |
