# Supplementary material for: Increasing trends in the prevalence of prior cancer in newly diagnosed lung, stomach, colorectal, breast, cervical, and corpus uterine cancer patients: a population-based study
Source: BMC Cancer. 2021 Mar 10;21:264. doi: 10.1186/s12885-021-08011-3 (PMC7948331; doi:10.1186/s12885-021-08011-3)
Supplement: Supplementary file 4 — Additional file 4: Supplementary Table S4. Temporal trends in the proportion of smoking-related prior cancers among index cancer patients [file 12885_2021_8011_MOESM4_ESM.docx]

**Additional File 4**

**Supplementary Table S4.** **Temporal trends in the proportion of smoking-related prior cancers among index cancer patients**

|  | | | | | | | | | | | | |
| --- | --- | --- | --- | --- | --- | --- | --- | --- | --- | --- | --- | --- |
| Diagnostic year of index cancer | 2004–2005 | | 2006–2007 | | 2008–2009 | | 2010–2011 | | 2012–2013 | | 2014–2015 | |
|  | N | % | N | % | N | % | N | % | N | % | N | % |
| Smoking-related cancers |  |  |  |  |  |  |  |  |  |  |  |  |
| Male | 992 | 81.4 | 1,421 | 80.2 | 1,528 | 77.3 | 1,997 | 74.4 | 2,295 | 72.4 | 2,436 | 70.7 |
| Female | 354 | 52.4 | 530 | 55.7 | 588 | 53.8 | 711 | 52.9 | 865 | 53.7 | 1,013 | 56.2 |
| Total | 1,346 | 71.1 | 1,951 | 71.6 | 2,116 | 69.0 | 2,708 | 67.2 | 3,160 | 66.1 | 3,449 | 65.7 |
|  |  |  |  |  |  |  |  |  |  |  |  |  |
| Non-smoking-related cancers |  |  |  |  |  |  |  |  |  |  |  |  |
| Male | 227 | 18.6 | 352 | 19.9 | 448 | 22.7 | 687 | 25.6 | 876 | 27.6 | 1,010 | 29.3 |
| Female | 321 | 47.6 | 421 | 44.3 | 505 | 46.2 | 633 | 47.1 | 745 | 46.3 | 791 | 43.9 |
| Total | 548 | 28.9 | 773 | 28.4 | 953 | 31.1 | 1,320 | 32.8 | 1,621 | 33.9 | 1,801 | 34.3 |
| Total |  |  |  |  |  |  |  |  |  |  |  |  |
| Male | 1,219 | 100 | 1,773 | 100 | 1,976 | 100 | 2,684 | 100 | 3,171 | 100 | 3,446 | 100 |
| Female | 675 | 100 | 951 | 100 | 1,093 | 100 | 1,344 | 100 | 1,610 | 100 | 1,804 | 100 |
| Total | 1,894 | 100 | 2,724 | 100 | 3,069 | 100 | 4,028 | 100 | 4,781 | 100 | 5,250 | 100 |
